# Genomics of periodontal disease and tooth morbidity

**DOI:** 10.1111/prd.12320

**Published:** 2019-12-18

**Authors:** Thiago Morelli, Cary S. Agler, Kimon Divaris

**Affiliations:** ^1^ Department of Periodontology School of Dentistry University of North Carolina at Chapel Hill Chapel Hill North Carolina, USA; ^2^ Department of Oral and Craniofacial Health Sciences School of Dentistry University of North Carolina at Chapel Hill Chapel Hill North Carolina, USA; ^3^ Department of Pediatric Dentistry School of Dentistry University of North Carolina at Chapel Hill Chapel Hill North Carolina, USA; ^4^ Department of Epidemiology Gillings School of Global Public Health University of North Carolina at Chapel Hill Chapel Hill North Carolina, USA

**Keywords:** classification, dentistry, endophenotypes, genomics, precision, prediction

## Abstract

In this review we critically summarize the evidence base and the progress to date regarding the genomic basis of periodontal disease and tooth morbidity (ie, dental caries and tooth loss), and discuss future applications and research directions in the context of precision oral health and care. Evidence for these oral/dental traits from genome‐wide association studies first emerged less than a decade ago. Basic and translational research activities in this domain are now under way by multiple groups around the world. Key departure points in the oral health genomics discourse are: (a) some heritable variation exists for periodontal and dental diseases; (b) the environmental component (eg, social determinants of health and behavioral risk factors) has a major influence on the population distribution but probably interacts with factors of innate susceptibility at the person‐level; (c) sizeable, multi‐ethnic, well‐characterized samples or cohorts with high‐quality measures on oral health outcomes and genomics information are required to make decisive discoveries; (d) challenges remain in the measurement of oral health and disease, with current periodontitis and dental caries traits capturing only a part of the health‐disease continuum, and are little or not informed by the underlying biology; (e) the substantial individual heterogeneity that exists in the clinical presentation and lifetime trajectory of oral disease can be identified and leveraged in a precision medicine framework or, if unappreciated, can hamper translational efforts. In this review we discuss how composite or biologically informed traits may offer improvements over clinically defined ones for the genomic interrogation of oral diseases. We demonstrate the utility of the results of genome‐wide association studies for the development and testing of a genetic risk score for severe periodontitis. We conclude that exciting opportunities lie ahead for improvements in the oral health of individual patients and populations via advances in our understanding of the genomic basis of oral health and disease. The pace of new discoveries and their equitable translation to practice will largely depend on investments in the education and training of the oral health care workforce, basic and population research, and sustained collaborative efforts..

## 1 INTRODUCTION

The existence of innate susceptibility and heritability of oral health and disease traits is well‐established. In fact, scholarly work discussing possible hereditary components of dental caries was published as early as the 1920s.^1^ It was several decades later, and as a result of continued progress in the basic sciences, breakthroughs in the supporting technologies, and substantial investments in effort and resources, that dentistry began to enter the “genome era”. The first genome‐wide association studies of periodontitis and dental caries were published in 2010^2^ and 2011,^3^ respectively. Nevertheless, other lines of research (including investigations among twins and families, and many candidate‐gene studies) have helped to build a solid case for the putative role of genetic factors in periodontal disease and dental caries. Excellent reviews and comprehensive summaries of the body of evidence supporting heritable components of oral disease have been published previously.^4^, ^5^, ^6^, ^7^, ^8^, ^9^

Genomic investigations (ie, aiming to study the genome, as opposed to genetics, traditionally focused on individual genes) have been successful in identifying important susceptibility loci for several complex disorders, including diabetes, obesity, Parkinson's disease, and several forms of cancer.^10^ Arguably, the major benefits of the genome‐wide association study approach include its agnostic nature (ie, hypothesis‐free as opposed to a candidate‐gene design) and coverage of a substantial proportion of the common variants found in the human genome (ie, producing information on millions of single nucleotide polymorphisms). From a methodologic standpoint, it is crucial to note that most reported associations from candidate‐gene studies fail to replicate in subsequent agnostic genome‐wide association study scans. There are several reasons behind this phenomenon, including the frequent low statistical power of genome‐wide association studies. However, the false‐positive ratio and the probable publication bias in the candidate‐gene literature are arguably more important issues. Ioannidis and colleagues,^11^ in a comprehensive quantitative analysis of candidate‐gene association replication in the genome‐wide association study era, found that ~1%‐5% of previously reported candidate‐gene associations were subsequently replicated by genome‐wide association studies. From an evidence‐based dentistry standpoint, this is important to acknowledge, as most oral health genomics evidence to date has been derived from candidate‐gene studies.^12^

Recently, its low cost has been added to the list of benefits of the genome‐wide association study methodology; high‐density genotyping can be undertaken for less than $100 per participant. The value and potential of genome‐wide association studies are now amplified by the increasing availability of whole genome sequence data, which can be used as reference panels for the imputation of additional (usually rare) markers that have not been directly genotyped,^13^ using two‐step^14^ or other imputation approaches. Moreover, the growth of publicly available data on the functional or regulatory role of single markers and genes offers additional opportunities for the annotation and functional interpretation of genome‐wide association study results.^15^, ^16^, ^17^, ^18^, ^19^

The efficient interrogation of the large and frequently multi‐omics data structures that accompany genome‐wide association study‐based research is certainly not straightforward, but parallel developments and advances in methodologic approaches and bioinformatics tools have made genome‐wide association studies increasingly accessible. Benefitting from this omics revolution, several genomics studies have been conducted in the oral health domain during the last decade, offering novel insights into the genomics of periodontal disease and tooth morbidity. The field is arguably in its early stages. However, several successes and noteworthy findings have marked the last decade and there is reasonable expectation that this progress will help to inform the realization of precision oral health and care, ultimately resulting in better individual clinical outcomes and improved population health.^20^, ^21^, ^22^

In the following section we provide an overview of research findings from genomic investigations of periodontal disease, dental caries, and several other related intermediate or composite traits. We highlight key points from each line of investigation, summarize where the field stands, and what future directions and opportunities lie ahead. As noted above, comprehensive reviews of periodontal genetics or genomics have been recently provided by Nibali et al,^4^ Schaefer,^6^ and Vieira and Albandar.^7^

## 2 GENOMICS OF TRADITIONAL CLINICAL DEFINITIONS OF ORAL AND DENTAL DISEASE

### 2.1 Periodontal disease

The genome‐wide association study of aggressive periodontitis reported by Schaefer et al^2^ marked the field's entry into the genome era. In that study, the investigators discovered and subsequently replicated the association of rs1537415, located in the glycosyltransferase gene (*GLT6D1*), with aggressive periodontitis. More recently, Sanders et al^23^ reported a significant association of a relatively rare *TSNAX‐DISC1* noncoding RNA polymorphism (rs149133391) with chronic periodontitis among Hispanic/Latino people and subsequently replicated it among an independent sample of African‐Americans. Other studies^24^, ^25^, ^26^, ^27^, ^28^, ^29^ have implicated numerous loci without reaching genome‐wide statistical significance levels and/or demonstrating replication in an independent cohort. For example, a recent study among a small Italian population reported associations between *EFCAB4B* polymorphisms (rs242016 showing the strongest evidence of association) and localized periodontitis.^24^

Interestingly, several loci have been highlighted as showing suggestive evidence of association (with *P* values typically ranging between 5 × 10^−6^ and 5 × 10^−8^) in more than one (independent) genome‐wide association study and thus warrant attention. A prime example is *SIGLEC5* (rs12461706), which was reported in a recent study of aggressive periodontitis^30^ and was the only locus that met genome‐wide statistical significance criteria in a large, consortium meta‐analysis of chronic periodontitis that combined clinical and self‐reported data.^31^ In a recent Korean study, Hong et al^25^reported *TENM2* (*ODZ2*) as being putatively associated with chronic periodontitis, whereas an earlier genome‐wide association study by Divaris et al^32^ highlighted this locus for its association with *Aggregatibacter actinomycetemcomitans* subgingival colonization levels in a North American sample. Defensin alpha 1 and alpha 3 (*DEFA1A3*) polymorphisms (rs2978951 and rs2738058) have been reported by both a recent aggressive periodontitis study^30^ and an earlier chronic periodontitis study.^29^ *NPY* is another locus of interest. There was suggestive evidence of an association in a North American genome‐wide association study of chronic periodontitis^27^ and it was the locus with the strongest evidence of association in a German male‐only stratified sample of aggressive periodontitis.^33^ Additional genome areas with multiple, independent genome‐wide association level of evidence, albeit not reaching genome‐wide significance or formal genome‐wide replication, include *ANRIL*,^34^, ^35^, ^36^, ^37^, ^38^ *CAMTA1/VAMP3*,^32^, ^38^, ^39^ *PF4*/*PPBP*/*CXCL5*,^40^ *NIN*/*CDKL1*,^27^, ^41^ *PLG*,^27^, ^30^, ^42^, ^43^ *VAMP8* (rs1561198),^44^ *MTND1P5* (rs16870060), and LOC107984137/*SHISA9* (rs729876).^45^ A gene‐centric and gene set enrichment re‐analysis of our group's single‐marker genome‐wide association study of chronic periodontitis^27^ and periodontal pathogen colonization^32^ has also been reported, including variable definitions of “gene boundaries”.^46^ Six genes showed genome‐wide evidence of association, four with severe chronic periodontitis (*NIN*,* P* = 1.6 × 10^−7^; *ABHD12B*,* P* = 3.6 × 10^−7^; *WHAMM*,* P* = 1.7 × 10^−6^; *AP3B2*,* P* = 2.2 × 10^−6^) and two with high periodontal pathogen colonization (red complex‐*KCNK1*,* P* = 3.4 × 10^−7^; *Porphyromonas gingivalis*‐*DAB2IP*,* P* = 1.0 × 10^−6^). The top‐ranked genes for moderate chronic periodontitis were *HGD* (*P* = 1.4 × 10^−5^), *ZNF675* (*P* = 1.5 × 10^−5^), *TNFRSF10C* (*P* = 2.0 × 10^−5^), and *EMR1* (*P* = 2.0 × 10^−5^). Loci containing *NIN*,* EMR1*,* KCNK1*, and *DAB2IP* had showed suggestive evidence of association in the earlier single nucleotide polymorphism‐based analysis,^27^ whereas *WHAMM* and *AP2B2* emerged as novel candidates. Finally, a recent genome‐wide association study of lipopolysaccharide‐induced periodontitis in mice identified CXCR3 as a susceptibility locus for bone loss in that model.^47^

Other notable investigations have used genotyping arrays with exome content,^48^ whole‐exome sequencing,^49^, ^50^ RNA expression/multi‐omics profiling,^51^, ^52^ and several post hoc bioinformatics and machine learning classification approaches^53^, ^54^ to study the molecular and genomic basis of periodontitis. The study of gene expression and other more functional approaches (as opposed to genetic association studies) is a key complement to genome‐wide association studies. Of note, most signals and markers highlighted in genome‐wide association studies tend to be associated with regulatory areas of the genome.^55^ In a recent report, Kitagaki and colleagues^49^ used whole‐exome sequencing to identify *GPR126* (lead marker: rs536714306) as a candidate genetic risk factor for aggressive periodontitis in a Japanese population. In another recent investigation, Sudo and colleagues^50^ used a two‐step approach in a family based study and used whole‐exome sequencing to identify novel mutations in the *NOD2* gene, which is also associated with aggressive periodontitis.

Some of the limitations of genome‐wide association studies for periodontitis are related to the sample size of the study and the inherent complexity in defining the trait. A commonly cited limitation in the periodontal genome‐wide association study literature is the relatively modest sample sizes of individual studies compared with reports for other common complex diseases, ranging between a few hundred^24^, ^28^ and up to 10 000‐17 000 participants.^23^, ^26^ This has been somewhat overcome by the establishment of the Gene‐Lifestyle Interactions and Dental Endpoints consortium^56^ and a recent report of periodontitis and dental caries genome‐wide association studies among over half a million individuals.^31^ However, perhaps the greater limitation in studying this disease is the substantial variation in the clinical periodontal phenotypes and case definitions for periodontitis in the published reports of the genomics of periodontitis. Additionally, the recent 2017 world workshop on the classification of periodontal and peri‐implant diseases and conditions reclassified aggressive and chronic periodontitis into one entity (“periodontitis”);^57^ which is then further characterized based on a multidimensional system that includes stages (ie, severity classifiers) and grades (ie, information on biologic features, including progression rate and risk). No report has yet examined the implications of the new classification system on the discovery of susceptibility loci for the disease.

Additional sources of heterogeneity or potential bias in the reported literature are related to tooth loss, which is known to bias estimates of periodontitis in cross‐sectional studies. Cases of periodontitis may be unidentified because of tooth loss. In the extreme scenario of edentulism (ie, total tooth loss), periodontitis cannot be defined, with the possibility of missing severe or aggressive cases of periodontitis as a result of rapid tooth loss. Importantly, the risk and reasons for tooth loss may be population and study specific,^58^ possibly underlying the observed heterogeneity between genome‐wide association studies of periodontitis. 31 Classification systems that capture and operationalize patterns of tooth loss have been developed. Although they are useful vehicles for the harmonization of different periodontal cohorts and samples,^59^, ^60^ they have yet to be interrogated in the context of genome‐wide association studies.

Key points: The number of genetic loci associated with periodontal disease obtained from genome‐wide association studies of aggressive and chronic periodontitis is increasing. Few loci, mainly identified for aggressive and severe forms of the disease, have met genome‐wide statistical significance criteria and have been replicated in independent investigations. Most genome‐wide association studies of periodontitis have been based on moderate or small sample sizes and there is substantial heterogeneity in their studied populations, the methods used, and the results reported.

### 2.2 Dental caries

The first genome‐wide association study for dental caries, published in 2011, was carried out for “childhood caries”, namely dental caries lesions manifested in the primary dentition.^3^ A subsequent report comprising five independent cohorts^61^ investigated dental caries in the permanent dentition and was published 1 year later. None of these investigations detected significant genome‐wide signals, although several loci had suggestive evidence of an association or had emerged from stratified analyses. More recent genome‐wide association studies were conducted for adult dental caries^62^ and early childhood caries^63^ and, similar to the previous studies, reported no significant loci but there were several suggestive, plausible ones (eg, *NAMPT* and *BMP7* for adult dental caries). A recent consortium meta‐analysis of childhood caries^64^ reported two significant genome‐wide loci (*ALLC*, rs1594318 and *NEDD9*, rs7738851) as well as heterogeneity and low heritability (1%) in the measured trait, compared with individual studies or previously published estimates.^65^

Although not the traditional clinical definition, dental caries patterns (ie, groups of tooth surfaces, such as pits and fissures) and subtypes have been used, with slightly more noteworthy outcomes in the context of genome‐wide association studies. For example, significant genome‐wide signals were reported for *LYZL2* (rs399593) and *AJAP1* (rs3896439) for subtypes (ie, dental caries patterns) of adult dental caries,^66^ and for *KPNA4* (rs17236529) for pit‐and‐fissure dental caries lesions in the primary dentition.^67^ A similar genome‐wide association study investigating clusters of pit‐and‐fissure and smooth surface dental caries in the primary dentition did not identify any significant signals.^68^ Additional loci not meeting genome‐wide significance criteria but with multiple lines of supporting evidence requiring some attention, include *MMP16* (rs2046315 and rs10429371),^69^ *PKD2* (rs17013735, rs11938025, rs2725270) and *SIBLING* (rs2725233),^70^ *MPPED2* and *ACTN2,* 71 and *TRAV4*.^72^ Although additional insights have been gained by gene set enrichment re‐analyses of a dental caries genome‐wide association study reported by Wang et al^73^, the recent, large‐scale consortium meta‐analysis that included clinical and self‐reported data (including denture use) among half a million individuals, discovered 47 novel loci for adult dental caries.^31^

As is the case with periodontitis, there are substantial variations and methodologic areas for improvement in the reported genome‐wide association studies of dental caries, especially with clinical phenotype ascertainment. One such example pertains to the childhood disease domain, where primary teeth begin to shed after the age of 6 years, leading to the loss of potentially disease‐informative surfaces between the ages of 6 and 12 years (which is considered the age range of primary dentition caries). Additionally, dental restorative work (eg, fillings, crowns, dentures, and extractions) is known to inflate measures of dental caries burden compared with what would be measured among “untreated” individuals.^74^ Finally, and similar to studies of periodontitis, tooth loss and its cause (ie, dental caries, periodontitis, orthodontic reasons, trauma, or congenitally missing) may not always be discernable and will probably vary between populations and study samples.^58^

Key points: Genome‐wide association evidence of dental caries is still limited, but a few promising and plausible candidates with corroborating evidence have been reported. A recent, large‐scale, consortium meta‐analysis identified many loci associated with dental caries, providing a rich resource of candidates to be followed‐up in subsequent investigations. Nevertheless, sizeable genotyped cohorts with high‐quality clinical data, including detailed phenotypes of dental caries, are warranted.

## 3 GENOMICS OF COMPOSITE, INTERMEDIATE, AND BIOLOGICALLY INFORMED TRAITS OF ORAL HEALTH AND DISEASE

Clinical definitions of disease typically capture a subset of the disease process or its expression. Interestingly, case definitions and diagnostic criteria for both periodontitis and dental caries have evolved over time, mostly as a result of changes in their population prevalence and, to some degree, improvements in our understanding of the disease process.^75^, ^76^, ^77^, ^78^ For example, most genome‐wide association study evidence for periodontitis has been generated by studies that used clinical measures of probing depth and attachment loss, whereas varying thresholds and criteria have been applied to derive person‐level dental caries experience indices. These measures are very popular and understandable by both scientific and clinical audiences. However, they arguably fail to capture the biologic aspects of periodontitis and dental caries. Of note, both diseases are essentially of dysbiotic‐microbial nature and share common risk factors.^79^ In spite of these similarities, periodontitis is characterized by an aberrant inflammatory response to commensal and dysbiotic subgingival microbial communities, whereas dental caries is a sustained tooth surface‐supragingival biofilm dysbiosis that leads to progressive demineralization of the dental hard tissues. Of note, the measurement of both diseases is undermined to some degree by tooth loss, especially in cross‐sectional studies, where reasons for tooth loss may be unknown or unclear. In this section we outline the genomic investigations of composite (ie, comprising more than one disease), intermediate (ie, part of the disease process, typically including inflammation and microbiome), and biologically informed (ie, clinical traits enriched with information on biologic parameters, typically inflammation and microbiome). We demonstrate the potential benefits of these traits over conventional clinical taxonomies.

### 3.1 Tooth morbidity

**Figure 1:**
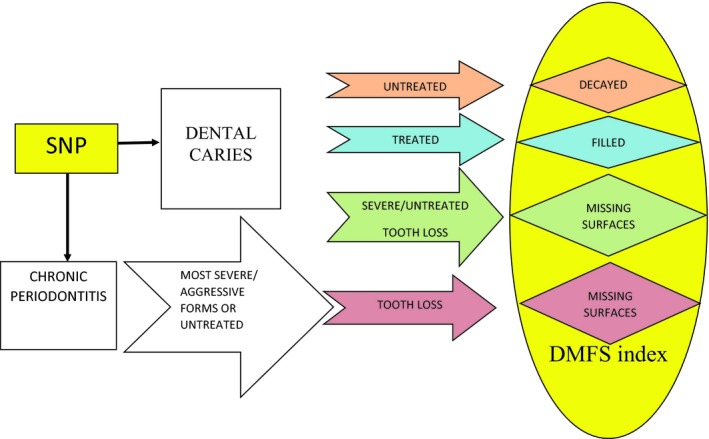
Theorized pathways contributing to the tooth morbidity (DMFS) index, emanating from dental caries and periodontitis. SNP, single nucleotide polymorphism

Tooth loss is the most common type of oral impairment and disability, with 79% of American adults aged 50 years and older having lost one or more teeth and 11% being edentulous.^80^ Apart from the obvious functional, biologic, and psychosocial consequences, edentulism (partial and complete) is associated with substantial rehabilitation costs and affects quality of life. In terms of etiology, tooth loss is attributed predominantly to the two most common oral diseases, caries and periodontitis. Accurate estimates of the individual contributions of caries and periodontitis to tooth loss are lacking and are probably heterogeneous across populations and between study samples;^58^ these proportions are also probably affected by numerous factors, including age, diet, smoking, dental care, and others. Nevertheless, the two diseases share a common etiologic basis: they are associated with pathogenic shifts in the oral microbiome and their pathogenesis entails complex interactions of highly organized intra‐oral biofilms with host immunity and protective factors. As reviewed earlier, genome‐wide evidence regarding genetic risk loci in caries and periodontitis has emerged. However, little attention has been given to the examination of the joint effects of these diseases on the dentition. To address this knowledge gap, our group proposed a composite “tooth morbidity” index [the sum of decayed, missing due to all causes (ie, “total”), and filled surfaces; DM_T_FS], which captures the cumulative dental effects of caries and periodontitis (Figure 1) and has been interrogated in a genome‐wide association study context.^81^ Conceptually, this analysis can be considered analogous to a genome‐wide association study of all‐cause mortality.^82^

The genome‐wide association study was carried out on a sample of approximately 4500 European‐American participants (mean age = 62 years) of the Atherosclerosis Risk in Communities study.^83^ Genotyping was carried out using the Affymetrix 6.0 platform and imputation to 2.5 million markers was based on HapMap II‐CEU.^27^, ^81^ Dental examination data were used to construct the composite, tooth morbidity index (DM_T_FS), comprising decayed surfaces, missing surfaces (total, due to all causes), and filled surfaces (excluding third molars and range: 0‐128). The association between single nucleotide polymorphisms (minor allele frequency ≥ 5%) and DM_T_FS was estimated using linear regression models assuming additive genetic effects. Models were adjusted for age, sex, study examination center, and population stratification, and a conventional multiple‐testing correction (*P* < 5 × 10^−8^) was applied. Exploratory analyses included additional adjustment for smoking, diabetes, body mass index, and chronic periodontitis, and stratification by periodontitis diagnosis, ie, healthy/mild periodontitis, moderate periodontitis, and severe periodontitis.

**Figure 2:**
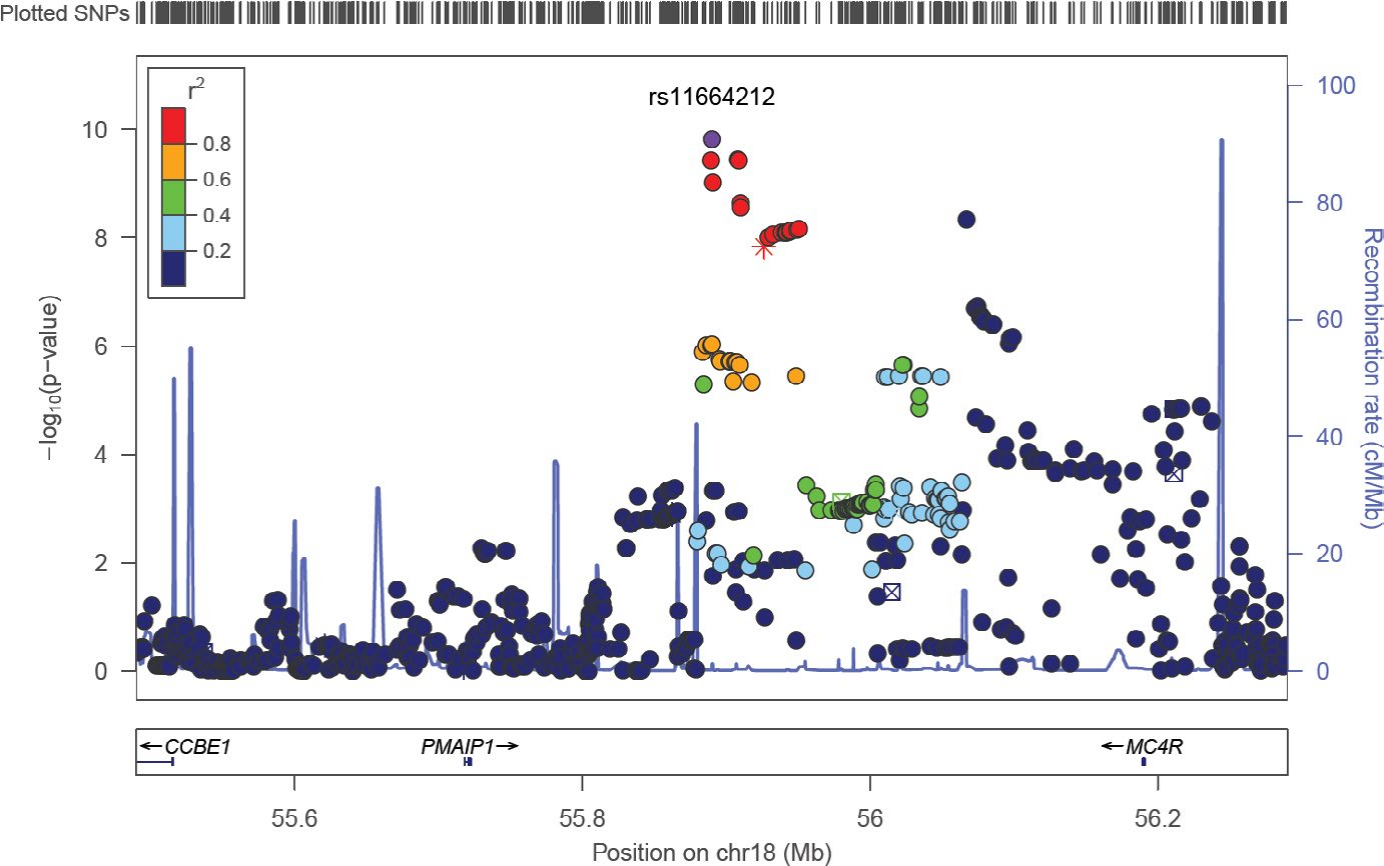
Regional association (Locus Zoom) plot of the PMAIP1/MC4R locus, which showed two independent association signals (rs11664212, minor allele frequency = 0.35, *P* = 1.5 × 10^−10^ and rs752720, minor allele frequency = 0.46, 3.1 × 10^−9^) in the genome‐wide association study of tooth morbidity in the dental Atherosclerosis Risk in Communities study. SNP, single nucleotide polymorphism

The mean DM_T_FS of the sample was 69 (standard deviation = 26). Four loci showed genome‐wide statistically significant evidence of an association with tooth morbidity (Table 1): *PMAIP1* (rs11664212; b = 3.65, *P* = 1.5 × 10^−10^), *SPC25* (rs477309; b = 4.23, *P* = 2.7 × 10^−9^), *MC4R* (rs752720; b = 3.74, *P* = 3.1 × 10^−9^), and *MPP7* (rs1262024; b = 5.80, *P* = 3.7 × 10^−8^). There was no evidence of heterogeneity (ie, effect measure modification) in periodontitis diagnosis‐stratified analyses. Of note, these associations persisted after adjustment for periodontitis diagnosis, smoking, diabetes, and body mass index (Table 2). These loci, with the exception of MPP7, also showed significant associations with the number of remaining natural teeth, but not with periodontitis diagnosis (Table 3), suggesting that dental caries is probably the driver of this association signal in this study population. Of these loci, *PMAIP1*/*MC4R* (Figure 2) is of particular interest, as it was subsequently reported with a significant genome‐wide signal for dental caries in the recent meta‐analysis of the Gene‐Lifestyle Interactions and Dental Endpoints consortium, which included over half a million individuals.^31^

**Table 1:** Genome‐wide association analysis results of tooth morbidity (number of decayed, missing, or filled tooth surfaces) among the dental Atherosclerosis Risk in Communities study participants, overall and stratified by chronic periodontitis diagnosis. The table presents the lead single nucleotide polymorphism (ie, the marker with the lowest *P* value) for each of the four loci that met genome‐wide significance criteria (*P* < 5 × 10^−8^ and minor allele frequency ≥5%) in the study sample

Chromsome	Locus	SNP	MAF^a^	Risk allele	Entire sample (n = 4398)			Chronic periodontitis diagnosis^b^								
								Healthy (n = 1785)			Mild/moderate (n = 1862)			Severe (n = 751)		
					Beta	SE	*P*	Beta	SE	*P*	Beta	SE	*P*	Beta	SE	*P*
18	PMAIP1	rs11664212	[G] 0.35	[G]	3.65	0.57	1.5 × 10^−10^	4.09	0.92	8.8 × 10^−6^	3.58	0.83	1.7 × 10^−5^	3.14	1.44	2.8 × 10^−2^
2	SPC25	rs477309	[T] 0.14	[C]	4.23	0.71	2.7 × 10^−9^	5.61	1.14	8.1 × 10^−7^	3.03	1.08	4.8 × 10^−3^	4.08	1.71	1.6 × 10^−2^
18	MC4R	rs752720	[T] 0.46	[C]	3.74	0.63	3.1 × 10^−9^	3.69	1.00	2.2 × 10^−4^	3.90	0.95	4.0 × 10^−5^	3.79	1.59	1.6 × 10^−2^
10	MPP7	rs1262024	[A] 0.08	[A]	5.80	1.05	3.7 × 10^−8^	6.18	1.64	1.6 × 10^−4^	4.01	1.63	1.3 × 10^−2^	8.50	2.58	9.2 × 10^−4^

Abbreviations: MAF, minor allele frequency; SE, standard error; SNP, single nucleotide polymorphism.

a Based on HapMap II‐CEU.

b All homogeneity X^2^ (test of between chronic periodontitis diagnosis strata heterogeneity) *P* > 0.2.

**Table 2:** Genome‐wide association analysis results of tooth morbidity (number of decayed, missing, or filled tooth surfaces) among the dental Atherosclerosis Risk in Communities study participants, “unadjusted” and adjusted for smoking and diabetic status, body mass index, and periodontitis diagnosis. The table presents the lead single nucleotide polymorphism (ie, marker with the lowest *P* value) for each of the four loci that met genome‐wide significance criteria (*P* < 5 × 10^−8^ and minor allele frequency ≥5%) in the entire study sample

Chromosome	Locus	SNP	MAF^a^	Risk allele	“Unadjusted” (n = 4398)			Fully adjusted models^b^								
								Chronic periodontitis diagnosis			Smoking, diabetes, body mass index			Smoking, diabetes, body mass index, and chronic periodontitis diagnosis		
					Beta	SE	*P*	Beta	SE	*P*	Beta	SE	*P*	Beta	SE	*P*
18	PMAIP1	rs11664212	[G] 0.35	[G]	3.65	0.57	1.5 × 10^−10^	3.66	0.57	1.2 × 10^−10^	3.48	0.56	6.9 × 10^−10^	3.47	0.56	7.8 × 10^−10^
2	SPC25	rs477309	[T] 0.14	[C]	4.23	0.71	2.7 × 10^−9^	4.25	0.71	2.3 × 10^−9^	4.29	0.70	1.0 × 10^−9^	4.22	0.70	2.0 × 10^−9^
18	MC4R	rs752720	[T] 0.46	[C]	3.74	0.63	3.1 × 10^−9^	3.75	0.63	2.8 × 10^−9^	3.48	0.63	2.7 × 10^−8^	3.50	0.63	2.2 × 10^−8^
10	MPP7	rs1262024	[A] 0.08	[A]	5.80	1.05	3.7 × 10^−8^	5.81	1.05	3.5 × 10^−8^	5.96	1.04	1.1 × 10^−8^	5.81	1.04	2.6 × 10^−8^

Abbreviations: MAF, minor allele frequency; SE, standard error; SNP, single nucleotide polymorphism.

a Based on HapMap II‐CEU.

b All changes in estimate after adjustment <10%.

**Table 3:** Association results of the four loci that were prioritized from the tooth morbidity genome‐wide association study with edentulousness and chronic periodontitis traits among the Atherosclerosis Risk in Communities study participants. *P* values were based on logistic regression models for the edentulous and chronic periodontitis traits, and a linear regression model for the number of remaining natural teeth

Chromosome	Locus	SNP	Edentulous vs dentate^a^ (n = 8103)	Number of natural teeth^b^ (0‐32) (n = 5538)	Moderate chronic periodontitis vs healthy^c^	Severe chronic periodontitis vs healthy^c^
18	PMAIP1	rs11664212	0.18	0.00049	0.21	0.22
2	SPC25	rs477309	0.97	0.0087	0.26	0.06
18	MC4R	rs752720	0.22	0.00082	0.53	0.17
10	MPP7	rs1262024	0.34	0.24	0.10	0.09

Abbreviation: SNP, single nucleotide polymorphism.

a Based on dental screening results among the Atherosclerosis Risk in Communities study participants.

b Based on a combination of dental screening and complete dental examinations among the Atherosclerosis Risk in Communities study participants.

c Based on comprehensive periodontal examinations among the dental Atherosclerosis Risk in Communities study participants.

Key points: Composite measures of oral disease offer promising and perhaps efficient alternatives to conventional and individual measures of dental caries and periodontitis. A genome‐wide association study of tooth morbidity among a moderately sized sample of European‐Americans identified four significant genome‐wide signals; one of these loci (PMAIP1/MC4R) was subsequently replicated in a consortium meta‐analysis.

### 3.2 The oral microbiome and inflammatory mediators

Understanding the genomic basis of clinical traits and directly observable health and disease end points is of natural interest to both clinicians and investigators. In the context of common complex diseases, such as periodontitis and tooth morbidity, it is expected that several loci contribute to disease development.^84^ Different loci and environmental factors are also probably involved, driving the disease incidence among different population subgroups. Theoretically, the presumably weak association signals of these loci should be detectable in clinical traits if large sample sizes are available. Another, alternative and complementary, approach is the interrogation of “biologic proximate” disease traits that are more likely to demonstrate a direct biologic connection with genetic loci. This approach has been successfully implemented in psychiatric and neurologic traits,^85^ where the observed traits can be very heterogeneous; these intermediate traits are often referred to as “endophenotypes”. This approach is conceptually analogous to the genetic interrogation of serum lipids (the endophenotypes) in the context of genetic studies of cardiovascular disease (the clinical end point).^86^

The composition and the function of the oral microbiome in states of oral health and disease^87^ are obvious target endophenotypes for both periodontitis and dental caries/tooth morbidity. To date, no exploration of the genomic basis of the supragingival (ie, dental caries‐related) microbiome composition has been undertaken; however, some evidence in the context of periodontitis exists. This line of investigation has been termed “infectogenomics” by Nibali and colleagues,^88^, ^89^, ^90^ and several plausible candidates associated with subgingival pathogen colonization have emerged from candidate‐gene studies (not reviewed here). The only genome‐wide association study of periodontal pathogen colonization, carried out by our group in 2012,^32^ did not detect any significant genome‐wide association signals in single marker (single nucleotide polymorphism) analyses. However, two loci showed evidence of a statistically significant genome‐wide association in subsequent gene‐centric re‐analyses (*KCNK1*,* P* = 3.4 × 10^−7^ for high “red complex” colonization and *DAB2IP*,* P* = 1.0 × 10^−6^ for *Porphyromonas gingivalis* high colonization).^45^ Moreover, three single nucleotide polymorphisms initially prioritized (*P* < 5 × 10^−6^) by our genome‐wide association study of periodontitis (rs2521634; *NPY* locus)^27^ and periodontal pathogen colonization (rs10010758; *TBC1D1* locus and rs10043775; *FBXO30* locus)^32^ were subsequently found to be associated with periodontal pathogen colonization by a recent independent study by Cavalla et al.^91^ Specifically, the *NPY* locus was associated with *Tannerella forsythia*,* Actinomyces gerencseriae*,* Fusobacterium periodonticum*, and *Prevotella nigrescens* colonization, the *TBC1D1* locus with *P. gingivalis*, and the *FBXO30* locus with *Prevotella intermedia*, after adjustment for multiple testing. Of note, both the genome‐wide association study and the subsequent candidate‐polymorphism study were carried out using DNA‐DNA checkerboard for the characterization of the subgingival microbiota. One can reasonably anticipate improvements in this line of host genomics investigation via the utilization of microbial community‐wide assessments (eg, whole genome sequencing shotgun or metagenomics), as well as more “functional” approaches (eg, RNA sequencing or metatranscriptomics).

Several lines of investigation have examined the host response and inflammation in the context of periodontitis and the discovery of their genomic basis is of great interest. From a clinical standpoint, a severe gingival inflammation index has been interrogated in the genome‐wide association study context.^92^ Our group identified a significant genome‐wide association signal in the *ASIC2* (formerly known as *ACCN1*) locus (lead marker: rs11652874; *P* = 3.9 × 10^−8^). However, this finding has not been replicated or mechanistically confirmed and should be treated with caution. From a more biologic standpoint, gingival crevicular fluid is an easily obtainable and highly informative biofluid that has been used as a marker of periodontal tissue inflammation and, thus, an endophenotype for periodontitis. The gingival crevicular fluid is a serum transudate that is modified by the local host response to the subgingival microbiome and it can serve as a biomarker of the microbial activation of the host's immune response. To date, gingival crevicular fluid interleukin‐1beta expression is the only periodontitis‐specific inflammatory endophenotype for which evidence of a genome‐wide association exists.^93^ Importantly, interleukin‐1beta has been established as a robust marker for severe inflammation, bone loss, and periodontal disease progression, and is known to be strongly genetically controlled. In brief, in a recent comprehensive genome‐wide association study and mechanistic follow‐up investigation, our group recently reported that variants in the *IL37* locus (lead marker: rs3811046) strongly controlled gingival crevicular fluid interleukin‐1beta expression (*P* = 3.3 × 10^−22^) and were also associated with 10‐year incident tooth loss and aggressive periodontitis assessed in an independent cohort. This investigation also showed a previously undetected heterogeneity in the genetic control of gingival crevicular fluid interleukin‐1beta expression. Specifically, we found that the *IL37* locus predominantly controlled (lead marker: rs3811046; *P* = 7.2 × 10^−20^) the high‐end of the distribution (eg, “top 10%” or profoundly hyper‐inflammatory vs bottom 50%) as opposed to the *IL1B* locus (lead marker: rs16944), which predominantly controlled (*P* = 3.2 × 10^−8^) mild elevations in interleukin‐1beta expression (eg, 50th‐75th percentile vs bottom 50%). This is a key finding in the search for the elements of the “hyper‐inflammatory trait”, as IL37, a member of the interleukin‐1 family of cytokines, is now being recognized as a natural suppressor of inflammatory and immune responses.^94^

Key points: Endophenotypes of oral diseases, mainly measures of the microbiome and the host response, are primary candidates for genomics studies in the context of periodontitis and dental caries/tooth morbidity. Although not always directly linked with a clinically measurable end point, these lines of investigation can uncover important biologic pathways that may otherwise be hard to detect via the study of heterogeneous clinical traits. Some genome‐wide evidence exists to support the genomic basis of both the microbiome and the host response in the context of oral health and disease.

### 3.3 Biologically informed, complex traits

A logical extension of the genomic interrogation of dental and periodontal endophenotypes is the combination of these biologic intermediates with clinical measures of health and disease, to create “biologically informed” complex traits. Therefore, our group recently combined clinical (ie, periodontal) and biologic (ie, subgingival periodontal pathogen colonization and gingival crevicular fluid interleukin‐1beta expression) data, using a principal components approach, to create six periodontal complex traits.^95^ This methodology has been used previously in genome‐wide association studies of complex facial morphology^96^ and bone traits.^97^

The six periodontal complex traits showed distinct and identifiable microbial and inflammatory profiles (eg, periodontal complex trait‐1 was characterized by a uniformly high pathogen load; periodontal complex trait‐3 showed high inflammatory and *A. actinomycetemcomitans* loading; and periodontal complex trait‐5 was dominated by *P. gingivalis*). Using these biologically informed, complex periodontal traits, we reported evidence of a genome‐wide association for 12 novel loci, specifically: periodontal complex trait‐1: *CLEC19A*,* TRA*,* GGTA2P*,* TM9SF2*,* IFI16*, and *RBMS3*; periodontal complex trait‐3: *C1QTNF7* and *TSNARE*; periodontal complex trait‐4: *HPVC1*; and periodontal complex trait‐5: *SLC15A4*,* PKP2*, and *SNRPN*. Some additional follow‐up evidence on the role of IFI16/AIM2 variants in periodontitis and their association with inflammatory and microbiologic parameters has since been reported by Marchesan et al.^98^

The biologic enrichment of these complex traits as well as the endophenotypes reviewed in the previous sections has obvious advantages in the search for associated genomic loci, as the traits are more proximal to biologic processes that are controlled by the genome. At the same time, the study of these traits comes with several limitations. A major limitation is related to the impossibility or difficulty in replicating genome‐wide association signals for these traits, as, thus far, they tend to be unique to the study samples they are created in (ie, the Atherosclerosis Risk in Communities study is the only one to have analyzed gingival crevicular fluid‐interleukin‐1beta expression and the subgingival microbiome in a clinical periodontitis cohort). Other limitations, also relevant to the traditional clinical disease definitions, are related to measurement, ie, differences under which studies are carried out and measurements are taken, and the known influence of tooth loss on periodontitis ascertainment.

Key points: The study of biologically informed, complex traits (ie, combining clinical, microbial, and host‐response information) has been the most productive approach to date for detecting genome‐wide signals and promising candidates for further mechanistic studies in periodontitis. However, these complex traits are virtually impossible to replicate in independent samples and have been generated from relatively small sample sizes. For these reasons, these findings should be treated with caution.

### 3.4 “Precision” periodontal traits

To address the limitations imposed by the influence of tooth loss on disease measurement, the need to facilitate clinical data harmonization across studies and the opportunity to capitalize on all available (eg, tooth‐level) clinical information, our group recently embarked upon a novel, latent class analysis^99^ approach to derive a new classification system for periodontitis.^59^, ^60^, ^100^ In brief, the approach is analogous to an unsupervised clustering procedure, where individuals and teeth are placed within mutually exclusive categories – periodontal profile classes and tooth profile classes, respectively. Our group identified seven distinct periodontal profile classes and seven distinct tooth profile classes that aid in patient stratification,^59^ are predictive of periodontitis progression and tooth loss,^60^ and are arguably better‐suited for precision oral health applications than current conventional disease classifications.^100^, ^101^ A genome‐wide association study of the periodontal profile class classification has not yet been undertaken. Nevertheless, the new classification has resulted in phenotypes that are familiar to clinicians who recognize patterns of missing teeth, areas of recession, diminished periodontal support, and other aspects of the dentition. Similar efforts, albeit not based on latent class analysis, have been undertaken in the dental caries domain: Shaffer and colleagues^102^ reported the use of principal component and factor analysis approaches to derive heritable and nonheritable patterns of dental caries (ie, total disease burden, pit‐and‐fissure, and smooth surface decay) in the permanent dentition.

Key points: The identification and operationalization of classes of patients or disease variance vectors are aligned with the notion of precision oral health and offer advantages over traditional disease taxonomies. Some evidence exists in the dental caries domains that certain disease subtypes may be more heritable than others. Additional research in the periodontitis domain is needed to understand whether the periodontal profile class/tooth profile class classification can lead to novel genomics insights or discoveries.

## 4 POTENTIAL UTILITY OF GENOME‐WIDE ASSOCIATION STUDY FINDINGS FOR THE CONSTRUCTION OF A PERIODONTITIS GENETIC RISK SCORE

Our genome‐wide association study of periodontitis^27^ (as defined by the Centers for Disease Control and Prevention/American Academy of Periodontology classification criteria) demonstrated that considerable proportions of the phenotypic variance can be explained by genome‐wide association study single nucleotide polymorphisms. This proportion can be interpreted as heritability and was 0.22 (standard error = 0.19) for severe periodontitis. Interestingly, the heritable variance increased to 0.52 (standard error = 0.35) when a genome × smoking interaction term was considered. Naturally, once risk loci and specific risk markers have been discovered and confirmed for a disease or condition, questions regarding their predictive ability, and ultimately utility, emerge. However, virtually no work has been carried out in the oral health domain to examine the utility and feasibility of using genome‐wide association study findings in the construction of informative periodontitis or dental caries “predictive” and risk models. Admittedly, risk assessment and outcome prediction using genome‐wide association study single nucleotide polymorphisms are categorically challenging for complex diseases,^103^ which tend to be polygenic and with a strong environmental or behavioral risk component. Additionally, the oral health field has not yet reached a consensus on validated and replicated genetic risk markers for dental caries and periodontitis. In spite of this, our group sought to demonstrate the potential utility of genome‐wide association study findings for the development and evaluation of periodontitis “predictive” models using sets of demographic, behavioral, and genetic factors prioritized from agnostic genome‐wide association study scans.

We used linkage disequilibrium pruning to identify 658 independent genomic loci that had a minor allele frequency >5%, an imputation quality score >0.8, and were associated at *P* < 0.001 with severe periodontitis.^104^ A logistic regression model for severe chronic periodontitis prediction was saturated after the inclusion of the top 287 markers; area under the curve = 0.998, subjects correctly classified = 98.5%, positive predictive value = 98.1%. In contrast, a model for moderate chronic periodontitis required almost double the number of prioritized genome markers to reach saturation (n = 481). Models including fewer numbers of markers also showed reasonable performance. For example, for the top 40 loci (Table 4): area under the curve = 0.866, subjects correctly classified = 81.5%, positive predictive value = 73.5%; for the top 100 loci: area under the curve = 0.929, subjects correctly classified = 87.7%, positive predictive value = 82.8%. The classification performance of all of these models clearly outperformed models wherein simulated, uninformative single nucleotide polymorphisms were used instead of those of the genome‐wide association study.

**Table 4:** The 40 most strongly associated (lowest *P* value in the discovery gemome‐wide association study of chronic periodontitis in the dental Atherosclerosis Risk in Communities study) loci with severe periodontitis, after linkage disequilibrium pruning. The top single nucleotide polymorphism in each locus is retained, with minor allele frequency >5% and imputation quality score >0.8

No.	Chromosome	Single nucleotide polymorphism rs id	Minor allele frequency	Effect allele
1	14	rs12883458	0.104	C
2	3	rs11925054	0.134	G
3	7	rs2521634	0.246	G
4	1	rs12073917	0.128	G
5	15	rs2890313	0.217	C
6	7	rs2106737	0.067	C
7	20	rs271972	0.481	A
8	21	rs7281463	0.414	C
9	5	rs16889923	0.128	A
10	1	rs11577771	0.127	C
11	7	rs1688605	0.122	G
12	11	rs10765844	0.478	G
13	4	rs1534582	0.213	G
14	4	rs17006135	0.071	C
15	11	rs10790919	0.212	A
16	21	rs2410204	0.406	A
17	7	rs258920	0.145	A
18	20	rs6029598	0.127	C
19	6	rs7772901	0.213	A
20	17	rs2871289	0.377	C
21	6	rs12175557	0.077	A
22	6	rs9791329	0.302	A
23	11	rs1398282	0.451	C
24	6	rs4485988	0.054	C
25	7	rs4527765	0.139	A
26	2	rs11695297	0.372	A
27	10	rs4751326	0.324	C
28	4	rs6814571	0.469	C
29	6	rs2305089	0.485	C
30	12	rs10771435	0.460	C
31	5	rs10043322	0.290	C
32	18	rs4800313	0.213	T
33	12	rs12314141	0.110	A
34	7	rs1541363	0.112	T
35	14	rs8008037	0.331	A
36	11	rs12364480	0.213	T
37	9	rs373637	0.429	C
38	15	rs4238336	0.231	C
39	16	rs4270178	0.500	A
40	12	rs10444531	0.236	T

**Figure 3:**
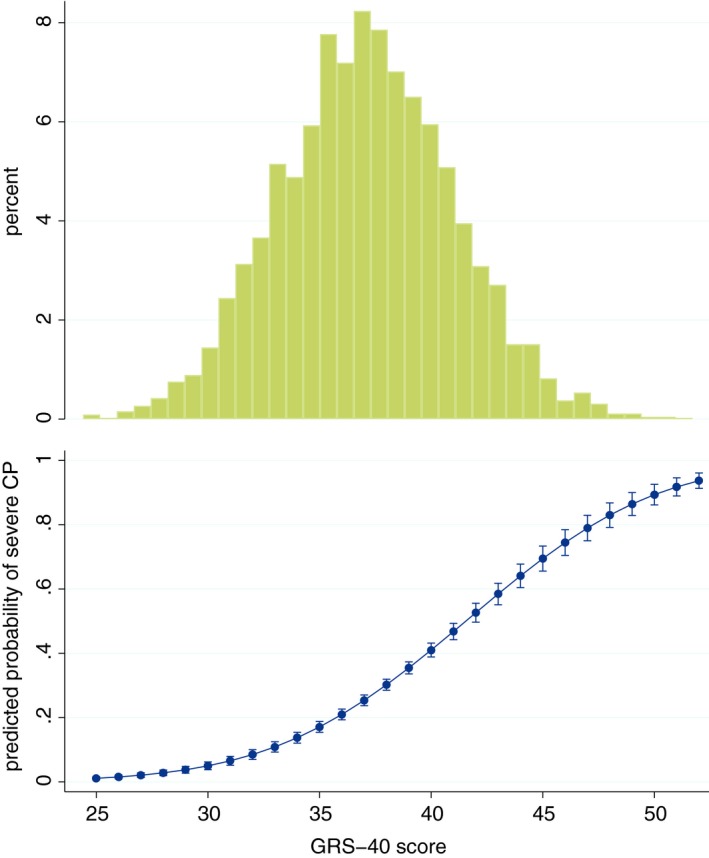
Distribution of the genetic risk score (GRS)‐40 index (top panel), together with the predicted probability (and 95% confidence intervals) of severe periodontitis (vs healthy/mild, according to the Centers for Disease Control and Prevention/American Academy of Periodontology classification) in the dental Atherosclerosis Risk in Communities study sample (bottom panel), derived from the genome‐wide association study of chronic periodontitis (CP)

**Figure 4:**
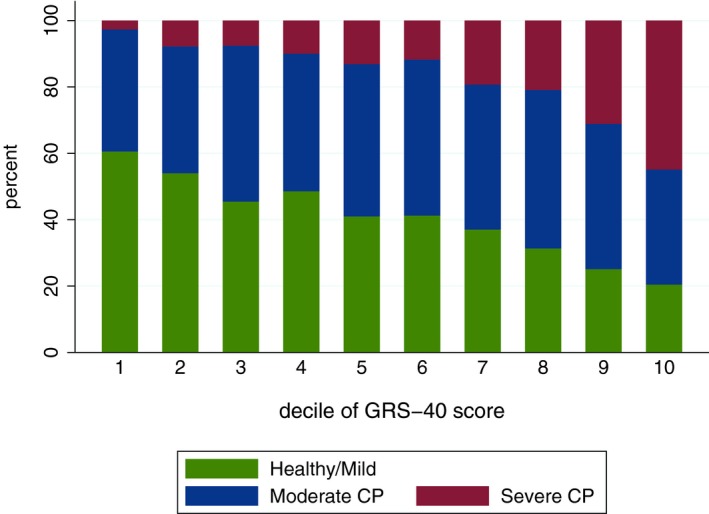
Distribution of periodontitis diagnoses (healthy/mild, moderate and severe chronic periodontitis, according to the Centers for Disease Control and Prevention/American Academy of Periodontology classification criteria) according to decile‐categories of the genetic risk score (GRS)‐40 index, derived from the genome‐wide association study of chronic periodontitis (CP)

We further examined the distribution and properties of the summary score of the “top 40” set of markers, ie, the genetic risk score‐40 (Figure 3, top panel). Its theoretical range is 0‐80; in our sample it was normally distributed with median = 37.1, mean = 37.1 (standard deviation = 3.9), range = 24.4‐51.7; there was no difference between sexes and no association with the age of the participants. As expected, the score was strongly positively associated with the model‐predicted probability of a participant having severe periodontitis (Figure 3, bottom panel). When categorized in deciles, the score also correlated well with periodontitis diagnoses in the entire dental Atherosclerosis Risk in Communities study sample (Figure 4).

We caution that the genetic risk score‐40 is by no means a validated, predictive, genetic score for periodontitis propensity, for several reasons. First, the genetic markers used were discovered in only one cohort and do not represent validated or replicated risk indicators. The markers and the risk score itself must be examined for replication in independent cohorts. Second, the discovery genome‐wide association study was conducted among a sample of middle‐age European‐Americans and for a specific clinically defined taxonomy of chronic periodontitis; both of these features severely limit the transferability of these findings to other populations and settings. Third, factors other than genomics are known to play important roles in the development of oral disease (eg, behavioral risk factors, socioeconomic environment, access to oral health care, to name a few). Therefore, although these indices (upon validation and replication) may provide useful information that is true “on average” and at the population level, they will not always work at the individual level. A more comprehensive approach, taking into consideration individual susceptibility, environment, and behaviors, and operationalizing disease subtypes aligned with the notion of precision medicine,^20^, ^21^, ^101^ will probably be effective. In spite of these limitations, the work presented here demonstrates that once a consensus set of validated and replicated genomic markers for periodontitis, dental caries, or tooth morbidity is developed, the genetic association signals can be efficiently combined to create aids for oral health and disease risk assessment and individual susceptibility estimation. In the future, this approach can ultimately be informative for screening, prevention, therapy, and the overall management of oral health and disease.

Key points: Genomics association information gained from genome‐wide association studies can be efficiently combined in risk models and indices that are predictive of health and disease case statuses. We are far from reaching a consensus with regard to specific genomic risk markers that are validated and replicated for their association with oral disease. However, once these markers have been discovered, validated, and replicated, genetic risk scores can be developed and will be useful in the context of precision oral health. This approach will probably be informative for the more severe disease categories, where genomics presumably plays a greater role.

## 5 SUMMARY AND CONCLUSIONS

Various groups have identified few loci associated with aggressive and severe forms of periodontal disease. However, most genome‐wide association studies of periodontitis have been based on moderate or small sample sizes and there is substantial heterogeneity in the studied populations, the methods used, and the results reported. In respect to dental caries, genome‐wide association evidence is still limited, but a recent, large‐scale, consortium meta‐analysis has identified many loci associated with dental caries, providing a rich resource of candidates to be followed‐up in subsequent investigations. Nevertheless, sizeable genotyped cohorts with high‐quality clinical data including detailed phenotypes on dental caries are warranted.

Biologically informed composite traits of oral diseases, including the microbiome and the host response, offer alternatives to conventional classifications of diseases that have traditionally measured the history of disease experience. This allows the identification of different biologic endophenotypes that may have overlapping clinical presentations. It has been the most productive approach to date to detect genome‐wide significant association signals and promising candidates for further mechanistic investigations in periodontitis. However, these complex traits have their own limitations and should be interpreted with caution.

The application of precision medicine in oral health has introduced the opportunity to use data‐driven and biologically informed phenotypes in the management of oral health and disease. On the research side, we expect that this will also facilitate clinical data harmonization across studies and will offer ample opportunities to maximize the usable clinical information.

We conclude that exciting opportunities lie ahead to improve the oral health of individual patients and populations via advances in our understanding of the genomic basis of oral health and disease. The pace of new discoveries and their equitable translation to practice will largely depend on investments in the education and training of the oral health care workforce, basic and population research, and sustained collaborative efforts.
